# Características clínicas y microbiológicas de las infecciones urinarias en el primer año postrasplante renal

**DOI:** 10.31053/1853.0605.v80.n4.41244

**Published:** 2023-12-26

**Authors:** Paula Vilella, Juan Pablo Maldonado, María Fernanda Flores, Camila Debernardi, Karen Vilte Velasquez, Daniela Verónica Hernández, Pehuen Fernández, Jorge de La Fuente, Emanuel José Saad

**Affiliations:** 1 Hospital Privado Universitario de Córdoba Córdoba Argentina; 2 Instituto Universitario de Ciencias Biomédicas de Córdoba (IUCBC) Córdoba Argentina

**Keywords:** trasplante de riñón, infecciones urinarias, infecciones, huésped inmunocomprometido, kidney transplantation, urinary tract infections, infections, immunocompromised host, transplante de rim, infecções urinárias, infecções, inflamação, hospedeiro imunocomprometido

## Abstract

**Introducción:**

Las infecciones del tracto urinario (ITU) constituyen la infección más frecuente en los trasplantados renales (TR). El objetivo principal fue determinar las características clínicas y microbiológicas de las ITU que ocurren durante el primer año posterior al TR.

**Metodología:**

Estudio de cohorte retrospectivo, donde se incluyeron pacientes mayores 18 años que recibieron un TR entre 2009-2020 en dos hospitales de la ciudad de Córdoba. A través del registro en las historias clínicas se realizó seguimiento de los pacientes durante el primer año postrasplante y se analizaron los que presentaron al menos un episodio de ITU.

**Resultados:**

En el período de estudio, se realizaron 568 TR, de los cuales 207(36,4%) tuvieron al menos un episodio de ITU. En total hubo 419 episodios de ITU, 6(1,4%) episodios de ITU polimicrobianos, se identificaron un total de 426 microorganismos en total en los urocultivos. Del total de episodios 206(49,2%) ocurrieron entre los 31-180 días postrasplante. El principal agente etiológico fue *E. coli* con 225 aislamientos (52,8%) seguido de *Klebsiella sp* . con 94(22,1%). El 52,1% del total de episodios fueron causados por microorganismos multirresistentes (MOR). Entre los aislamientos de *E. coli* , 94(41,8%) fueron MOR. En el análisis multivariado los factores de riesgo asociados a ITU por MOR fueron el antecedente de ITU recurrente (Odds ratio 2.43; IC95%: 1.37-4.30) e inducción con basiliximab (Odds ratio 1.53; IC95%: 1.029-2.29).

**Conclusión:**

Las ITU se presentaron en más de un tercio de los pacientes trasplantados renales siendo un poco más de la mitad causados por MOR.

## Abstract (en)

**Introduction:**

Urinary tract infections (UTI) are the most frequent infection in kidney transplant (KT) patients. The main objective was to determine the clinical and microbiological characteristics of UTIs that occur during the first year after KT.

**Methods:**

Retrospective cohort study that included patients over 18 years of age who received KT between 2009-2020 in two hospitals in Córdoba city, Argentina. Patients were followed up during the first year after the transplant by recording them in the medical records, and those who presented at least one episode of UTI were analyzed.

**Results:**

During the study period, 568 KTs were performed, of which 207 (36.4%) had at least one episode of UTI. In total, there were 419 UTI episodes, 6 (1.4%) polymicrobial UTI episodes, so a total of 426 microorganisms were identified in the urine cultures. Of the total number of episodes, 206 (49.2%) occurred between 31 and 180 days post-transplant. The main etiological agent was *E. coli* with 225 isolates (52.8%) followed by *Klebsiella sp* . with 94 (22.1%). 52.1% of all episodes were caused by multi-resistant microorganisms (MRM). Among the *E. coli* isolates, 94 (41.8%) were MRM. In the multivariate analysis, the risk factors associated with UTI due to MRM were a history of recurrent UTI (Odds ratio 2.43; 95% CI: 1.37-4.30) and induction with basiliximab (Odds ratio 1.53; 95% CI: 1.029-2.29).

**Main conclusion:**

UTIs occurred in more than a third of kidney transplant patients, being slightly more than half caused by MOR.

## Resumo

**Introdução:**

As infecções do trato urinário (ITUs) são as infecções mais comuns em pacientes transplantados renais (TR). O objetivo principal foi determinar as características clínicas e microbiológicas das ITUs que ocorrem durante o primeiro ano após a TR.

**Métodos:**

Estudo de coorte retrospectivo que incluiu pacientes maiores de 18 anos que receberam TR entre 2009 e 2020 em dois hospitais da cidade de Córdoba. Os pacientes foram acompanhados durante o primeiro ano após o transplante por meio de registro em prontuário e analisados aqueles que apresentaram pelo menos um episódio de ITU.

**Resultados:**

Durante o período do estudo, foram realizados 568 TRs, dos quais 207 (36,4%) tiveram pelo menos um episódio de ITU. No total, houve 419 episódios de ITU, 6 (1,4%) episódios de ITU polimicrobiana, portanto, um total de 426 microrganismos foram identificados nas culturas de urina. Do total de episódios, 206 (49,2%) ocorreram entre 31 e 180 dias pós-transplante. O principal agente etiológico foi *E. coli* com 225 isolados (52,8%) seguido de *Klebsiella sp* . com 94 (22,1%). 52,1% de todos os episódios foram causados por microrganismos multirresistentes (MOR). Na análise multivariada, os fatores de risco associados à ITU por MOR foram história de ITU recorrente (Odds ratio 2,43; IC 95%: 1,37-4,30) e indução com basiliximab (Odds ratio 1,53; IC 95%: 1,029-2,29).

**Conclusão:**

ITUs ocorreram em mais de um terço dos pacientes transplantados renais, sendo pouco mais da metade causadas por MOR.

**CONCEPTOS CLAVE**

**Qué se sabe sobre el tema**

- Las infecciones urinarias (ITU) constituyen las infecciones más frecuentes en pacientes trasplantados renales.
- Con el paso de los años se encuentra aumentando la frecuencia de ITU por microorganismos multirresistentes (MOR).

**Qué aporta este trabajo**

- Un tercio de los pacientes trasplantados renales presentaron al menos un episodio de ITU en el primer año postrasplante.
- Más de la mitad de los aislamientos microbiológicos de los episodios de ITU fueron secundarios a MOR.
- El principal factor de riesgo asociado a desarrollo de ITU por MOR fue el antecedente de ITU recurrente.

**Divulgación**

Las infecciones urinarias (ITU) constituyen las infecciones más frecuentes en pacientes trasplantados renales, representando de esta manera una problemática de gran relevancia, más aún en un contexto actual de aumento de infecciones por microorganismos multirresistentes a antibióticos (MOR). En nuestro estudio hemos podido observar que un tercio de los pacientes trasplantados renales presentaron al menos un episodio de ITU en el primer año postrasplante y más de la mitad de los aislamientos microbiológicos fueron secundarios a MOR. El principal factor de riesgo asociado a desarrollo de ITU por MOR fue el antecedente de ITU recurrente.

## Introducción

El trasplante renal (TR) es considerado la mejor opción terapéutica para los pacientes con enfermedad renal crónica terminal (ERCt), ya que no solo mejora la calidad de vida, sino que también reduce la mortalidad con respecto a los pacientes que persisten en tratamiento de reemplazo renal con diálisis ^1-3^ . Sin embargo, a pesar de los grandes avances en técnicas quirúrgicas y terapéuticas inmunosupresoras, el receptor de un TR está expuesto a un riesgo de complicaciones infecciosas que pueden aumentar la morbimortalidad de los mismos, principalmente en los primeros 180 días, debido al estado de inmunosupresión ^4, 5^ . Entre los posibles factores de riesgo de infecciones se encuentran ciertas comorbilidades, infecciones latentes, severidad de la enfermedad que motiva el trasplante, duración de la estancia hospitalaria posterior al procedimiento quirúrgico, utilización de antibióticos, necesidad de utilización de terapia de reemplazo renal y reintervenciones quirúrgicas entre otras ^5-7^ .

Las infecciones del tracto urinario (ITU) constituyen la infección más frecuente en los pacientes con TR ^8, 9^ . Se reconoce que cerca del 70% es causada por bacterias Gram negativas ^10, 11^ , siendo *Escherichia Coli* el principal patógeno, con incrementos progresivos de su resistencia a los antimicrobianos con el paso de los años según registros de diferentes áreas geográficas ^11-13^ . Esto ha afectado a toda la población en general, incluso a pacientes sin factores de riesgo tan importantes como el caso de TR ^14^ . Se ha reportado en países de Latinoamérica y el caribe que durante el 2020-2021 posteriormente al inicio de la pandemia por COVID-19 un aumento de incidencia de infecciones por Enterobacterias productoras de carbapenemasa a un ritmo mucho mayor que lo observado previamente ^15^ . Todo esto ha motivado a organizaciones gubernamentales a tomar conductas con el objetivo de retrasar o impedir la emergencia y diseminación de bacterias resistentes, que por ejemplo en Argentina actualmente han llegado a plasmarse en la sanción en agosto de 2022 de la "Ley de Prevención y control de la resistencia a los antimicrobianos, Ley 27680" ^16, 17^ . Se trata de una ley pionera en Argentina, a nivel regional e incluso a nivel mundial, que tiene un abordaje en salud -humana, animal, ambiental-, que involucra a todas las áreas del Estado que trabajan en ese sentido ^17^ .

El conocimiento de la epidemiología local de las ITU en los pacientes TR, es relevante debido a la incidencia de dicha patología, el aumento progresivo de microorganismos multirresistentes (MOR) y la importante morbimortalidad asociada, con el objetivo de poder establecer estrategias de terapéuticas adecuadas a la situación epidemiológica ^5,10, 12^ . La información reciente sobre las características de las ITU en pacientes TR en el medio local es escasa, por lo que se plantea la necesidad de realizar una valoración de la misma.

El objetivo principal del estudio fue determinar las características clínicas y microbiológicas de los episodios de ITU en el primer año postrasplante renal durante el período de 2009-2020 en el Hospital Privado Universitario de Córdoba y la Unidad Sanatorial Raúl Ángel Ferreyra, de la ciudad de Córdoba en Argentina.

El objetivo secundario fue evaluar los posibles factores de riesgo de desarrollo de ITU por MOR en pacientes con TR en el primer año postrasplante.

## Materiales y Métodos

### Muestreo y recolección de datos

Se realizó un estudio observacional descriptivo retrospectivo en dos hospitales de tercer nivel de la Ciudad de Córdoba, el Hospital Privado Universitario de Córdoba y la Unidad Sanatorial Raúl Ángel Ferreyra. Los procedimientos de TR son desarrollados en el primero, mientras que el seguimiento de los pacientes se realiza de manera coordinada en ambos centros, que presentan los mismos equipos médicos tratantes. Posteriormente al TR los pacientes realizan controles periódicos frecuentes, siguiendo un protocolo institucional, permitiendo un seguimiento adecuado de los mismos.

Ambas instituciones comparten el mismo sistema de historia clínica electrónica a partir de los cuales se identificaron todos los pacientes mayores de 18 años que han sido receptores de TR en el período comprendido entre 01/01/2009 y 31/12/2020. Se excluyeron aquellos que hayan presentado trasplante de otro órgano sólido en el mismo período. Se revisaron las historias clínicas de los pacientes incluidos, registrándose información referente a los datos demográficos, comorbilidades, información referida al TR, complicaciones o invasiones pos-trasplante y todos los episodios de ITU ocurridas durante el seguimiento. Finalmente se incluyeron solo los pacientes TR que presentaron al menos 1 episodio de ITU durante el primer año pos-trasplante. El seguimiento de los pacientes se realizó hasta el momento en que se cumpliese un año postrasplante, se sometiera a nuevo trasplante de órgano sólido, muriese o se perdiera su seguimiento, lo que haya ocurrido primero. Cabe destacar que para cada paciente trasplantado se ha realizado el análisis de factores de riesgo para ITU con respecto al primer episodio de ITU que haya presentado. Por otro lado, para evaluar los espectros de susceptibilidad antimicrobiana y factores de riesgo asociados a ITU por MOR, se han tomado en cuenta la totalidad de los aislamientos microbiológicos.

### Definiciones:

- Paciente TR:
  Aquel que haya recibido injerto renal de donante vivo o donante cadavérico con utilización concomitante de terapia inmunosupresora. Se evaluó el tipo de inmunosupresión utilizada como inducción y mantenimiento, reintervención quirúrgica post trasplante.
- Retraso de la función renal post trasplante del injerto:
  Necesidad de diálisis dentro de la primera semana después del trasplante ^18^ .
- ITU baja:
  Bacteriuria clínicamente significativa (>10^5^ UFC/ml, o >10^2^ UFC/ml en muestra de orina recolectada luego de inserción de un catéter) con síntomas de disuria, sin sensibilidad ni dolor en la proximidad del riñón trasplantado, con o sin deterioro de la función renal del injerto ^19^ . Asimismo, se ha considerado ITU baja a la presencia de bacteriuria en paciente trasplantado sin síntomas en el primer año pos-trasplante ^20^ .
- ITU alta
  Bacteriuria clínicamente significativa (>10^5^ UFC/ml, o >10^2^ UFC/ml en muestra de orina recolectada luego de inserción de un catéter), fiebre de >38°C y/o sensibilidad o dolor en la proximidad del implante renal y/o deterioro de la función renal y/o analítica con elevación de parámetros inflamatorios (proteína C reactiva o leucocitosis) y/o imagen renal o biopsia compatible con pielonefritis ^19^ .
- Infección Urinaria asociada a catéter (ITU-C)
  El paciente debía de cumplir los siguientes 3 criterios: 1) Tener un catéter urinario que había sido colocado al menos 2 días calendario antes de la fecha del evento (se define como día 1 al día en que se ha colocado el catéter), y el catéter ha permanecido colocado el día calendario del evento o ha sido removido el día previo al evento. 2) al menos uno de los siguientes signos o síntomas: fiebre mayor a 38° C, dolor abdominal (sin otra causa aparente), dolor o sensibilidad en el ángulo costovertebral (sin otra causa aparente); 3) urocultivo positivo con no más de 2 especies de microorganismos, al menos uno de ellos que desarrollen más de 10^5^ UFC/mL ^21^ .
- ITU recurrente:
  2 episodios de ITU no complicada en los últimos 6 meses o 3 urocultivos positivos en el año anterior ^19^ .

**Según el lugar donde fuesen adquiridas las ITU, se las clasificó en 2 grupos:**

- Infección adquirida en la comunidad:
  Cuando las manifestaciones clínicas o evidencia infectológica de la misma se desarrollaron de modo ambulatorio o hasta dentro de las primeras 48 horas de hospitalización, no mediando durante ese período ninguna actividad asistencial que pueda haberla inducido.
- Infecciones asociadas a la atención de la Salud (IAAS):
  Definida como toda infección que no esté presente o que no se incube en el momento del ingreso o que aparezca después del alta hospitalaria o el procedimiento médico ambulatorio. Puede ser causada directamente por la acción del microorganismo o a través de sus toxinas. Se ha considerado a ITU asociada a la atención de la salud, si la misma se ha manifestado al menos 48 horas después de la admisión o hasta 72 horas posteriores al alta de la misma ^19, 22^ .
- Microorganismos multirresistentes (MOR):
  Definidos a partir de los conceptos propuestos por el Centro de Control y Prevención de Enfermedades (CDC) y el Centro Europeo para la Prevención y el Control de Enfermedades (ECDC), considerando a una bacteria como multirresistente si esta es resistente al menos a un fármaco en tres o más categorías de fármacos antimicrobianos de relevancia para cada especie. En el caso de los bacilos Gram negativos, se los considera como multirresistentes si no son sensibles a tres de las siguientes categorías: penicilinas antipseudomónicas, cefalosporinas, carbapenémicos, aminoglucósidos o fluoroquinolonas ^23, 24^ . Entre los microorganismos Gram positivos, *Staphylococcus* resistentes a la meticilina y *Enterococcus* resistentes a la vancomicina son considerados patógenos multirresistentes ^23, 25^ .

El Laboratorio de Microbiología utilizó como método de rutina los sistemas automatizados VITEK 2 Compact (bioMérieux, Francia) y Phoenix 100 (Becton Dickinson, EE.UU.) para determinar la susceptibilidad antimicrobiana y espectrometría de masas MALDI-TOF Microflex (Bruker, Alemania) para la identificación de especie. El laboratorio se encuentra integrado al programa de control de calidad externo de pruebas de susceptibilidad antimicrobiana e identificación del Instituto de Salud ANLIS Dr. Carlos Malbrán. Se relevaron los espectros de resistencia a los antibióticos más frecuentemente utilizados en el internado.

### Análisis Estadístico:

Las variables continuas se expresaron como media y desviación estándar, o mediana y rango intercuartilo (RIC), y la comparación de las mismas se realizó con *test t de Student* o *U de Mann-Whitney* de acuerdo a su distribución. Las variables categóricas se expresaron como número y porcentaje y se compararon con *test de chi cuadrado* o exacto de Fisher, de acuerdo con las frecuencias esperadas. Se consideró como significativo un valor de probabilidad <0,05. Para los factores de riesgo de ITU por GMR se utilizó el riesgo relativo (RR) con su intervalo de confianza del 95% (IC95%). El análisis estadístico se realizó con el programa estadístico SPSS 24.0 (SPSS, Inc., Chicago, IL).

El estudio fue aprobado por el comité de investigación del Departamento de Docencia e Investigación del Hospital Privado Universitario de Córdoba, de Córdoba Argentina.

## Resultados

En el período comprendido entre 1 de enero de 2009 y el 31 de diciembre 2020 se desarrollaron 568 trasplantes renales únicos en pacientes de 18 años o mayores. De ellos, 207 presentaron al menos un episodio de ITU en el primer año postrasplante (de los cuales 112 [54,11%] tan sólo 1 episodio de ITU a lo largo del seguimiento). La mediana de meses de seguimiento fue de 12 meses (RIC= 12-12 meses), llegando a completar el seguimiento de 12 meses 181 (87,4%) pacientes. Entre los que no llegaron al año de seguimiento, 16 (7,7%) fallecieron, 9 (4,3%) tuvieron pérdida de seguimiento y 1 (0,5%) recibió un nuevo trasplante de órgano sólido antes de dicho período. Asimismo, durante el período de seguimiento se identificaron un total de 419 episodios de ITU, con 426 aislamientos microbiológicos, de los cuales 222 (52,1%) fueron causados por MOR (IC95%= 47.3-57.8%) (figura 1).

**Figura N° 1.**
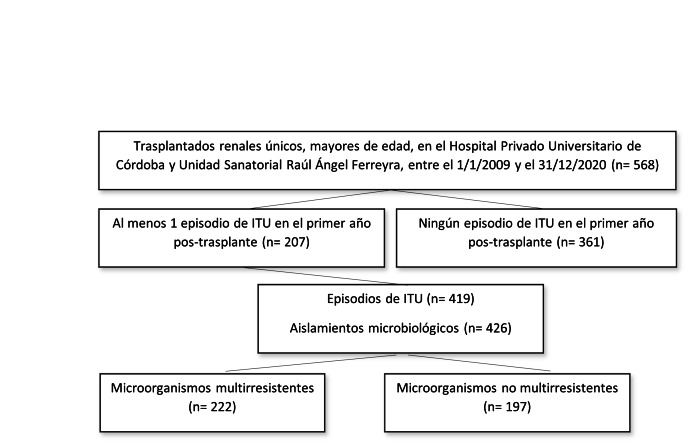
Diagrama de pacientes trasplantados renales incluidos

En la tabla 1 se muestran las características basales y relacionadas al trasplante de los pacientes que presentaron al menos un episodio de ITU durante el seguimiento. La mediana de edad fue de 53 años (RIC= 40-65 años) con predominancia de pacientes de sexo femenino (n=110, 53,1%). El 21.7% eran diabéticos, el 11,1% presentaban el antecedente de cardiopatía isquémica y 11,7% de vasculitis. El 11,1% había recibido inmunosupresión previa al trasplante y el 10,1% presentaban un trasplante previo. Las etiologías de la ERC más frecuentes fueron: desconocida (25,6%), glomerulonefritis primaria (16,4%) y nefropatía diabética (16,4%). Como inmunosupresión de inducción todos los pacientes recibieron esteroides, el 42% basiliximab y 33,8% timoglobulina. Como inmunosupresión de mantenimiento casi la totalidad de pacientes utilizaron esteroides (99,5%), tacrolimus (99%) y micofenolato (94,2%). El 67,1% de los pacientes recibió un trasplante de donante cadavérico. El 57% requirió de la colocación intraoperatoria de un catéter urinario pigtail, el 16.4% requirió de una reintervención quirúrgica, el 42,5% evolucionó con retraso de la función del injerto y el 5,3% como no funcionantes primarios. Durante el seguimiento y previo al episodio de ITU, el 47,8% (n=99) de los pacientes habían sido sometidos a un procedimiento invasivo de la vía urinaria y el 13% (n=27) había requerido de una sonda vesical en el mes previo.

**Tabla N° 1:** Características clínicas de los pacientes con infección urinaria (ITU) en el primer año post-trasplante renal (TR).

Características de los pacientes TR con ITU	Total (n=207)
Edad, Mediana (RIC)	53 (40-65)
Sexo femenino, n (%)	110 (53,1)
Comorbilidades	
Diabetes Mellitus, n (%)	45 (21,7)
Insuficiencia cardiaca, n (%)	13 (6,3)
Cardiopatía isquémica, n (%)	23 (11,1)
Lupus eritematoso sistémico, n (%)	10 (4,8)
Vasculitis, n (%)	49 (11,7)
Asma / EPOC, n (%)	15 (7,2)
Tabaquismo, n (%)	15 (7,2)
Virus de la inmunodeficiencia humana, n (%)	2 (1)
Trasplante previo, n (%)	21 (10,1)
Inmunosupresión previa, n (%)	25 (11,1)
Etiología de la enfermedad renal crónica	
Glomerulonefritis primaria, n (%)	34 (16,4)
Nefropatía diabética, n (%)	34 (16,4)
Nefroangioesclerosis, n (%)	22 (10,6)
Uropatía obstructiva, n (%)	17 (8,2)
Poliquistosis autosómica dominante, n (%)	15 (7,2)
Nefritis lúpica, n (%)	8 (3,9)
ERC de otra causa, n (%)	24 (11,6)
Desconocidos, n (%)	53(25,6)
Inmunosupresión de Inducción	
Esteroides, n (%)	207 (100)
Basiliximab, n (%)	87 (42)
Timoglobulina, n (%)	70 (33,8)
Gammaglobulina, n (%)	37 (17,9)
Plasmaféresis, n (%)	14 (6,8)
Inmunosupresión de Mantenimiento	
Esteroides, n (%)	206 (99,5)
Tacrolimus, n (%)	205 (99)
Micofenolato, n (%)	195 (94,2)
Azatioprina, n (%)	7 (3,4)
Inhibidores mTOR, n (%)	4 (1,9)
Características de Trasplante	
Donante Cadavérico, n (%)	139 (67,1)
Complicaciones o invasiones	
Colocación de Pigtail, n (%)	118 (57)
Reintervención quirúrgica, n (%)	34 (16,4)
Retraso de la función del injerto, n (%)	88 (42,5)
Trasplantado no funcionante primario, n (%)	11 (5,3)

*RIC:* Rango intercuartil; *EPOC:* Enfermedad pulmonar obstructiva crónica; *ERC: E* nfermedad renal crónica

Entre los 207 pacientes, existieron 419 episodios de ITU en total en total. La mayoría ocurrieron en el período entre los 31-180 días postrasplante (n=206, 49,2%), observándose 327 (78%) episodios de ITU alta, y siendo más frecuentemente adquiridas en la comunidad (n=334, 79,7%). Se destaca que 248 (59,2%) de los episodios de ITU tenían el antecedente de ITU en el año previo ( Tabla 2 ). De los 419 episodios, hubo 6 (1,4%) aislamientos polimicrobianos, por lo que se identificaron un total de 426 microorganismos en los urocultivos. Se observó una predominancia de bacterias bacilo Gram negativas (n= 394, 92,5%) correspondiendo 225 (52,8%) a *Escherichia coli* , de los cuales 94 (41,8%) fueron MOR, y 81 (36%) productores de betalactamasas de espectro extendido (BLEE). Otra de las bacterias más frecuentemente aisladas fue *Klebsiella sp* . con 94 (22,1%) aislamientos, de los cuales 71 (75,5%) fueron MOR y 69 (73,5%) productoras de BLEE, y 4 (4,26%) productoras de carbapenemasas. La bacteria Gram positivas más frecuente fue *Enterococcus* *faecalis* con 17 (4%) aislamientos. Los perfiles de susceptibilidad antimicrobiana de los principales aislamientos microbiológicos se detallan en la **Tabla 3** . Se puede observar un alto porcentaje de resistencia a trimetoprima/sulfametoxazol de *E. coli* (94,6%), *Klebsiella* (96,8%) y *Enterobacter* (96.7%).

**Tabla N°2:** Tipos y tiempo de aparición de los episodios de infección urinaria (ITU) en el primer año post trasplante renal.

Características de los episodios de ITU	ITU (n=419)
Tipo de infección	
Adquirida en la comunidad, n (%)	334 (79,7)
Infección asociada a atención de la salud, n (%)	85 (20,3)
ITU alta, n (%)	327 (78)
ITU baja, n (%)	92 (22)
Urosepsis, n (%)	65 (15,5)
Infección asociada a catéter, n (%)	58 (13,8)
Antecedente de ITU en el año previo, n (%)	248 (59,2)
Antecedente de ITU recurrente, n (%)	179 (42,7)
Tiempo desde trasplante a la infección	
0-30 días, n (%)	105 (25,1)
31-180 días, n (%)	206 (49,2)
181-365 días, n (%)	109 (26)
Microorganismos aislados (n= 426)	
Hongos, n (%)	4 (0,9)
*Candida sp* , n (%)	4 (0,9)
Bacterias Gram negativos *,* n (%)	394 (92,5)
*Escherichia coli,* n (%)	225 (52,8)
*Klebsiella sp, n* (%)	94 (22,1)
*Enterobacter sp,* n (%)	30 (7)
*Pseudomonas sp,* n (%)	14 (3,3)
Bacterias Gram positivos, n /%)	28 (6,6)
*Enterococcus faecalis* , n (%)	17 (4)

**Tabla N°3:** Perfil de susceptibilidad antimicrobiana de principales aislamientos microbiológicos de los episodios de infección urinaria en el primer año post trasplante renal.

Microorganismos identificados	Aislamientos no sensibles a antimicrobianos
Escherichia coli, n=225	
- Microorganismo multirresistentes, n(%)	94 (41,8)
- BLEE, n(%)	81 (36)
- Ampicilina/sulbactam, n/N(%)	145/224 (64,7)
- Cefalosporina 1 generación, n/N(%)	119/222 (53,6)
- Cefalosporina 3 generación, n/N(%)	88/223 (39,5)
- Cefepime, n/N(%)	81/224 (36,2)
- Ciprofloxacina, n/N(%)	135/224 (60,3)
- Trimetoprima/sulfametoxazol, n/N(%)	212/224 (94,6)
- Gentamicina, n/N(%)	53/221 (24)
- Imipenem, n/N(%)	1/224 (0,4)
- Piperacilina tazobactam, n/N(%)	24/217 (11,1)
- Amikacina, n/N(%)	1/223 (0.4)
*Klebsiella sp* . , n=94	
- BLEE, n(%)	69 (73,4)
- Productora de carbapenemasas, n(%)	4 (4,26)
- Microorganismo multirresistentes, n(%)	71 (75,5)
- Ampicilina/sulbactam, n/N(%)	78/94 (83)
- Cefalosporina 1 generación, n/N(%)	78/94 (83)
- Cefalosporina 3 generación, n/N(%)	69/92 (75)
- Cefepime, n/N(%)	69/93 (74,2)
- Ciprofloxacina, n/N(%)	64/94 (68,1)
- Gentamicina, n/N(%)	52/93 (55,9)
- Piperacilina tazobactam, n/N(%)	50/93 (53,8)
- Trimetoprima/ sulfametoxazol, n/N(%)	91/94 (96,8)
- Imipenem/ Meropenem, n/N(%)	5/93 (5.4)
Enterococcus faecalis, n= 17	
- Microorganismo multirresistentes, n(%)	3 (17,6)
- Ampicilina, n/N(%)	3/16 (18,8)
- Gentamicina, n/N(%)	0/17 (0)
- Vancomicina, n/N(%)	3/17 (17,6)
- Ciprofloxacina, n/N(%)	7/16 (43,8)
Enterobacter sp, n= 30	
- Microorganismo multirresistentes, n(%)	29 (96,7)
- BLEE, n(%)	27 (90)
- Ampicilina/sulbactam, n/N(%)	30/30 (100)
- Cefalosporina 1 generación, n/N(%)	29/29 (100)
- Cefalosporina 3 generación, n/N(%)	27/28 (96,4)
- Cefepime, n/N(%)	27/30 (90)
- Ciprofloxacina, n/N(%)	28/30 (93,3)
- Trimetoprima/sulfametoxazol n/N(%)	29/30 (96,7)
- Gentamicina, n/N(%)	28/30 (93,3)
- Imipenem, n/N(%)	0/30 (0)
- Piperacilina tazobactam, n(%)	20/30 (66,7)
- Amikacina, n/N(%)	8/30 (26,7)
*n/N(%)* : Aislamientos resistentes/total de testeos de dicho antibiótico, *BLEE:* Betalactamasas de espectro extendido.	

Al comparar la proporción de aislamientos de microrganismos productores de BLEE en los períodos de tiempo, se ha observado una mayor proporción en el período 2015-2020 respecto al 2009-2014 (53,7% -130/242 ITU- vs. 29,5%-51/173 ITU, p<0,01). De la misma manera el número de aislamientos de *E. coli* productora de BLEE fue mayor en el segundo período (47,2%, 58/123 ITU vs. 20,8% -20/96 ITU, p<0,01) ( Fig 2) .

**Figura N° 2.**
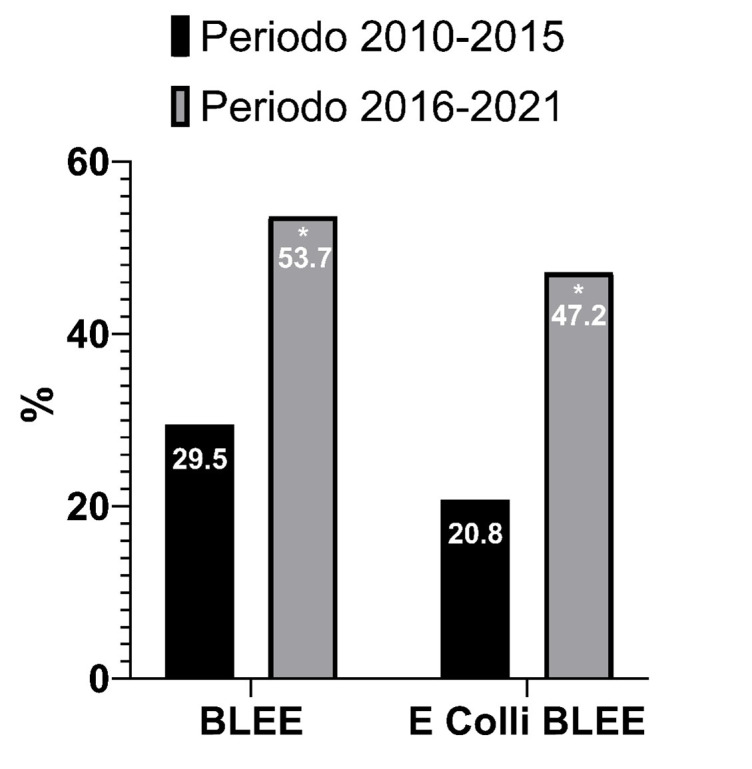
Porcentaje de infecciones urinarias por microorganismos productores de betalactamasas de espectro extendido (BLEE) en pacientes trasplantados en el primer año postrasplante renal.

Al comparar la proporción de aislamientos de microrganismos productores de BLEE en los períodos de tiempo de mayor número de episodios de ITU, se ha observado una mayor proporción en el período 1 de Enero de 2015 al 31 de diciembre de 2020 respecto al período de 1 de enero de 2009 al 31/12/2014 (53,7% -130/242 ITU- vs. 29,5%-51/173 ITU, p<0,01). Del mismo modo el número aislamientos de E. coli productora de BLEE fue mayor en el segundo período (47,2%, 58/123 ITU vs. 20,8% -20/96 ITU, p<0,01).

Entre los posibles factores de riesgo asociados al desarrollo de ITU por MOR en los pacientes TR se destacaron: antecedente de ITU recurrentes (52,7% vs 31,4%; p<0,001); ITU previa (65,8% vs 51,5%; p= 0,003) e inducción con basiliximab (65,3% vs 53.4%; p=0,013). Al realizar el análisis multivariado las variables que continuaron siendo significativas fueron ITU recurrente (Odds ratio 2.43; IC95%: 1.37-4.30) e inducción con basiliximab (Odds ratio 1.53; IC95%: 1.029-2.29). Los gérmenes que en mayor proporción son MOR son bacterias gran negativas, diferentes a la *E. coli* , como la *Klebsiella sp* . y *Enterobacter sp* . Los TR con antecedentes de ITU recurrentes tuvieron 62% mayor riesgo de presentar MOR comparado con los que tuvieron episodios de ITU no recurrente (RR=1,62; IC95%=1,29-2,02; p<0,001). No se hallaron diferencias estadísticamente significativas en relación a las comorbilidades, etiología de la ERC, inmunosupresión de mantenimiento, características del trasplante, invasiones o tipo de infección ( Tabla 4) . En un segundo análisis de sensibilidad, observamos que los pacientes que durante el seguimiento presentaron más de un episodio de ITU tuvieron 62% mayor riesgo de presentar MOR comparado con los que tuvieron un solo episodio durante el seguimiento (RR=1.62; IC95%=1.25-2.10; p<0,001). Además, por cada episodio nuevo de ITU durante el seguimiento, se incrementa un 20% las chances de presentar MOR (OR=1.20; IC95%=1.06-1.36; p=0,003).

**Tabla N° 4:** Factores asociados al desarrollo de infección urinaria por microorganismos multirresistentes (MMR) en el primer año postrasplante renal.

Variables	Con MMR (n=222)	Sin MMR (n=204)	p
Edad, Mediana (RIC)	57 (40-66)	50 (36-63)	0,18
Sexo femenino, n (%)	118 (53,1)	102 (50)	0,51
Comorbilidades			
Diabetes Mellitus, n (%)	49 (22,1)	36 (17,6)	0,25
VIH, n (%)	3 (1,3)	2 (1)	1
Trasplante previo, n (%)	24 (10,8)	32 (15,7)	0,14
Inmunosupresión previa, n (%)	21 (9,5)	26 (12,7)	0,28
Etiología de la enfermedad renal crónica			
Glomerulonefritis primaria, n (%)	17 (12,2)	33 (16,2)	0,23
Nefropatía diabética, n (%)	39 (17,6)	27 (13,2)	0,22
Uropatía obstructiva, n (%)	21 (9,5)	27 (13,2)	0,22
Inmunosupresión de Inducción			
Basiliximab, n (%)	77 (34,7)	95 (46,6)	0,01
Timoglobulina, n (%)	66 (29,7)	72 (35,3)	0,22
Gammaglobulina, n (%)	32 (14,4)	28 (13,7)	0,84
Características de Trasplante			
Donante Cadavérico, n (%)	161 (72,5)	141 (69,1)	0,44
Complicaciones o invasiones			
Colocación de Pigtail, n (%)	120 (54,1)	108 (52,9)	0,82
Reintervención quirúrgica, n (%)	33 (14,9)	33 (16,2)	0,71
Retraso de la función del injerto, n (%)	100 (45)	79 (38,7)	0,19
Trasplantado NFP, n (%)	5 (2,2)	7 (3,4)	0,46
Tipo de infección			
Adquirida en la comunidad, n (%)	173 (77,9)	167 (81,9)	0,31
IAAS, n (%)	49 (22,1)	37 (18,1)	0,31
Urosepsis, n (%)	38 (17,1)	27 (13,2)	0,27
Infección asociada a catéter, n (%)	34 (15,3)	24 (11,8)	0,29
ITU en el año previo, n (%)	146 (65,8)	105 (51,5)	0
ITU recurrente, n (%)	117 (52,7)	64 (31,4)	<0,001
Tiempo desde trasplante a la infección			
0-30 días, n (%)	51 (23)	57 (27,9)	0,24
31-180 días, n (%)	126 (56,8)	84 (41,2)	0
181-365 días, n (%)	46 (20,7)	63 (30,9)	0.002
Microorganismos aislados			
Bacterias Gram negativos, n (%)	213 (95,9)	181 (88,7)	0,01
*Escherichia coli,* n (%)	94 (42,3)	131 (64,2)	<0,001
*Klebsiella sp* , n (%)	71 (32)	23 (11,3)	<0,001
*Enterobacter sp,* n (%)	29 (13,1)	1 (0,5)	<0,001
*Pseudomonas sp,* n (%)	4 (1,8)	10 (4,9)	0,07
Bacterias Gram positivos, n /%)	9 (4)	19 (9,3)	0,03
*Enterococcus faecalis,* n (%)	2 (0,9)	15 (7,3)	0
*VIH* : Virus de la inmunodeficiencia humana; *NFP* : no funcionante primario; *IAAS* : Infección asociada a atención de la salud			

## Discusión

En el presente estudio se ha observado que un poco más de un tercio de los pacientes trasplantados renales han desarrollado al menos un episodio de infecciones urinarias en el primer año postrasplante, siendo el principal aislamiento microbiológico responsable *Escherichia coli* . Respecto al porcentaje de pacientes que han desarrollado ITU fue similar al observado por otros estudios donde la frecuencia fue cercana al 44,2% ^9^ .

Es de destacar que la mayoría de los episodios de ITU (49,2%) ocurrieron entre los 31 y 180 días postrasplante, período que se caracteriza por ser uno de los de mayor inmunosupresión de los pacientes trasplantados. Asimismo, este período se caracterizó por presentar mayor cantidad de ITU por MOR respecto a los otros períodos. Esto podría deberse a la fuerte influencia de los fármacos inmunosupresores, que incrementarían el riesgo de esta infección tan común en pacientes con alteración de la vía urológica y su recurrencia podría asociarse a incremento de resistencias antimicrobianas, como ha sido descripto previamente en otras publicaciones ^26-27^ . Respecto a aquellos factores de riesgo asociados al desarrollo de ITU por MOR en estudios previos, se ha destacado que aquellos con mayor influencia han sido el sexo femenino del receptor, donante cadavérico y la invasión de la vía urinaria previo a la infección ^28-29^ . El antecedente de donante cadavérico podría constituirse como un factor de riesgo debido a contribuir a mayor alteración de la vía urinaria, más aún si el paciente se le ha colocado catéter pigtail, siendo factores riesgo a tener en cuenta en estos escenarios para el desarrollo de ITU ^30^ . En nuestro estudio, estas variables no obtuvieron significancia estadística.

En la literatura se ha descripto que la diabetes mellitus se suele asociar a mayor riesgo de infecciones en pacientes trasplantados y mucho más aún en lo que respecta a ITU ^9^ . Sin embargo, existen otros estudios al igual que nuestro donde no se ha observado una asociación significativa entre esta comorbilidad y el desarrollo de ITU por MOR ^31^ . La invasión de la vía urinaria por el uso de catéter pigtail pos-trasplante y el uso de sonda vesical el mes previo en el presente estudio no presentaron aumento significativo del riesgo de ITU por MOR. Sin embargo, es reconocida que la utilización de catéter urinario permanente durante períodos mayores a los 10 días es un claro factor de riesgo independiente para adquirir ITU y la prolongación en el tiempo de uso del catéter urinario está directamente relacionada con el desarrollo de dicha infección ^32^ .

Se destaca que durante el período de estudio el 59,2% de los episodios de ITU habían tenido al menos 1 episodio de ITU en el año previo y el 42,7% cumplían con la definición de haber tenido ITU recurrentes en los meses previos, constituyendo porcentaje mayor a los encontrados en otros estudios ^33^ . Esta observación realizada de que un elevado porcentaje de antecedentes de ITUs previas, asimismo se vio asociado de manera significativa con el desarrollo de nuevos aislamientos en los urocultivos de MOR, posiblemente relacionado a la exposición a otros antimicrobianos en un pasado reciente.

El microorganismo más frecuente hallado fue *Escherichia coli* , dato que coincide con la bibliografía existente, constituyendo por el momento el principal agente causal de ITU en la población general ^5, 31-32^ . Durante los últimos años, el aumento de la resistencia antimicrobiana ha generado serios debates a nivel mundial. La población receptora de TR se encuentra sometida a un fenómeno de presión de selección para cepas de microorganismos resistentes como posible consecuencia del uso de profilaxis antimicrobiana postrasplante, o infecciones recurrentes, en este estudio se determinó que 52,1% de los aislamientos de los urocultivos de pacientes con ITU fueron MOR, valores ligeramente más elevados que los relatados por otros estudios ^19, 33-37^ .

Resulta importante destacar el hecho de que se han producido múltiples aislamientos secundarios a bacterias productoras de BLEE, representando el 36% de los aislamientos de *Escherichia coli,* el 73,4% de *Klebsiella sp* y el 90% de las *Enterobacter sp* . Asimismo, se ha evidenciado que la proporción de microorganismos productores de BLEE, prácticamente se han duplicado en los últimos 6 años del estudio, observado dicho cambio especialmente en *E. coli* . Al comparar esta información con la publicada por la Red de vigilancia de resistencia bacteriana a los antimicrobianos WHONET-Argentina ha informado que desde el 2010 al 2022 la resistencia a Cefalosporinas de tercera generación se ha incrementado para *E. coli* en aislamientos totales en población en general desde 18% al 26,3% y a imipenem del 0,8% al 2,6% ^38^ . Por otro lado, para tener en cuenta, según lo publicado en el Mapa de resistencia antimicrobiana en el año 2022 publicado por la Red WHONET, se describe más específicamente que la resistencia de *E. coli* a cefalosporinas de 3° generación en urocultivos es del 16,2% pero con una resistencia en el área centro del país entre el 10 y 25%, siendo mucho mayor en los países del norte del país. Por otro lado, la resistencia de *E. coli a* carbapenémicos en urocultivos en el país es del 0,8% ^39^ .

Por otro lado, las resistencias reportadas en Argentina en 2010 y 2022 a cefalosporinas de tercera generación se han mantenido estables para aislamientos en general de *Klebsiella sp* (63% a 56,4%), *Enterobacter sp* (47% a 46,6%) y *Pseudomonas sp* (23% a 26,3%) ^38^ . En el reporte realizado por la Red WHONET en el año 2022 en Argentina, se ha observado que el 48,7% de los aislamientos en urocultivos de *Klebsiella pneumoniae* eran resistentes a cefalosporinas de 3° generación y el 24,1% a carbapenémicos ^39^ .

La importancia de los aislamientos de microorganismos productoras de BLEE incluso es mayor en caso de que el paciente presente bacteriemia asociada, debido a que se encuentra asociada a mortalidad elevada cuyos reportes de frecuencia es variada, pero con una media del 31% aproximadamente ^40^ . Se ha desarrollado un Score denominado INCREMENT, el cual entre sus variables que suman puntos incluye la edad mayor a 50 años (Odds ratio= 2,63; IC95%: 1,18–5,85; 3 puntos), infección por *Klebsiella sp* (Odds ratio= 2,08; IC95%: 1,21–3,58; 2 puntos), origen diferente a infección urinaria (Odds ratio= 3,6; IC95%: 2,02–6,44; 3 puntos), comorbilidad basal mortal (Odds ratio= 3,91; IC95%: 2,24–6,80; 4 puntos), Score Pitt >3 (Odds ratio= 3,04; IC95%: 1,69–5,47; 3 puntos), sepsis severa o shock séptico en la presentación (Odds ratio= 4,8; 95% CI: 2,72–8,46; 4 puntos) y tratamiento inicial inapropiado (OR = 2,47; IC95%: 1,58–4,63; 2 puntos). De esta manera predice una mortalidad con scores < 11 y ≥11 de 5,4% y 34,8% respectivamente ^41^ .

Es importante destacar que, si bien se han identificado tan sólo 4 episodios de ITU secundarias a *Klebsiella sp* productora de carbapenemasa, la relevancia de la misma es notoria, más aún debido al incremento exponencial observado de este tipo de resistencias en los últimos años ^15^ . Este aspecto ha sido aspecto ha sido aún más notorio desde el comienzo de la pandemia por COVID-19 a fines de 2019, donde en las primeras épocas existían recomendaciones que incluían el uso de antimicrobianos, además de necesidad de internaciones prolongadas en unidades de cuidados críticos de los pacientes cuadros severos, aumentando el riesgo de sobreinfección bacteriana ^15, 42-43^ . La emergencia de infecciones causadas por microorganismos resistentes a carbapenémicos constituye uno de los mayores problemas sanitarios a nivel mundial, más aún cuando los patógenos producen betalactamasas ^15^ . La Red Latinoamericana de Vigilancia de la Resistencia a los Antimicrobianos (ReLAVRA), ha reportado en esta región que la frecuencia en general de aislamientos productores de carbapenemasa previo al año 2010 era ocasional, observándose un ligero pero sostenido incremento entre 2010-2019, haciendo un quiebre posteriormente con un aumento mucho mayor ^15^ .

Entre las limitaciones del presente estudio se destaca principalmente el hecho de ser estudio no multicéntrico, con una muestra no aleatorizada, que no incluye pacientes pediátricos, y que probablemente no represente a la totalidad de los trasplantes renales, de diseño retrospectivo en lo que respecta a las dificultades de poder asegurar la completa evaluación de la totalidad de los episodios infecciosos desarrollados durante el período de seguimiento de los pacientes, principalmente en aquellos pacientes cuyos domicilios no se encontraban en la ciudad de Córdoba Capital, donde se encontraban los consultorios de seguimiento pos-trasplante. Sin embargo, es importante destacar, que los pacientes TR en este centro realizan un seguimiento estrecho durante el primer año, permitiendo una recolección de muchos datos relacionados a su evolución, con consultas frecuentes principalmente en el primer año pos-trasplante. Otro aspecto destacable del estudio es que sólo un porcentaje pequeño de los pacientes ha perdido el seguimiento en el hospital, permitiendo tener una muestra bastante significativa, con un gran número de pacientes trasplantados a lo largo de varios años.

## Conclusión

Los episodios de ITU en el primer año postrasplante renal constituyeron una frecuente causa de morbilidad de los pacientes TR, siendo producidas en más de la mitad de los casos por MOR, cuya frecuencia se encuentra incrementándose a ritmo acelerado en los últimos años, principalmente luego del advenimiento de la infección COVID-19, con aumento de microorganismos productores de BLEE y aparición de gérmenes productores de carbapenemasas. Los principales factores asociados al desarrollo de infección por MOR han sido el antecedente de ITU en el último año, especialmente si se trataba de ITU recurrente, asociándose posiblemente entre otros factores a la exposición a antimicrobianos previamente.

Este estudio permitió conocer las características clínicas-microbiológicas locales, que podrían ser de utilidad para el desarrollo de protocolos de actuación médica adecuada y oportuna en estos pacientes.
